# Supplementary material for: Partition complex structure can arise from sliding and bridging of ParB dimers
Source: Nat Commun. 2023 Jul 29;14:4567. doi: 10.1038/s41467-023-40320-y (PMC10387095; doi:10.1038/s41467-023-40320-y)
Supplement: Supplementary file 1 — Supplementary Information [file 41467_2023_40320_MOESM1_ESM.pdf]

Supplementary Information for

Partition complex structure can arise from sliding and bridging of ParB dimers

Lara Connolley[1], Lucas Schnabel[2], Martin Thanbichler[1,2], Seán M. Murray[1,*]

1. Max Planck Institute for Terrestrial Microbiology and Center for Synthetic Microbiology, 35043, Marburg, Germany
2. Department of Biology, University of Marburg, 35043, Marburg, Germany

∗ Corresponding author e-mail: sean.murray@synmikro.mpi-marburg.mpg.de.

Supplementary Figures

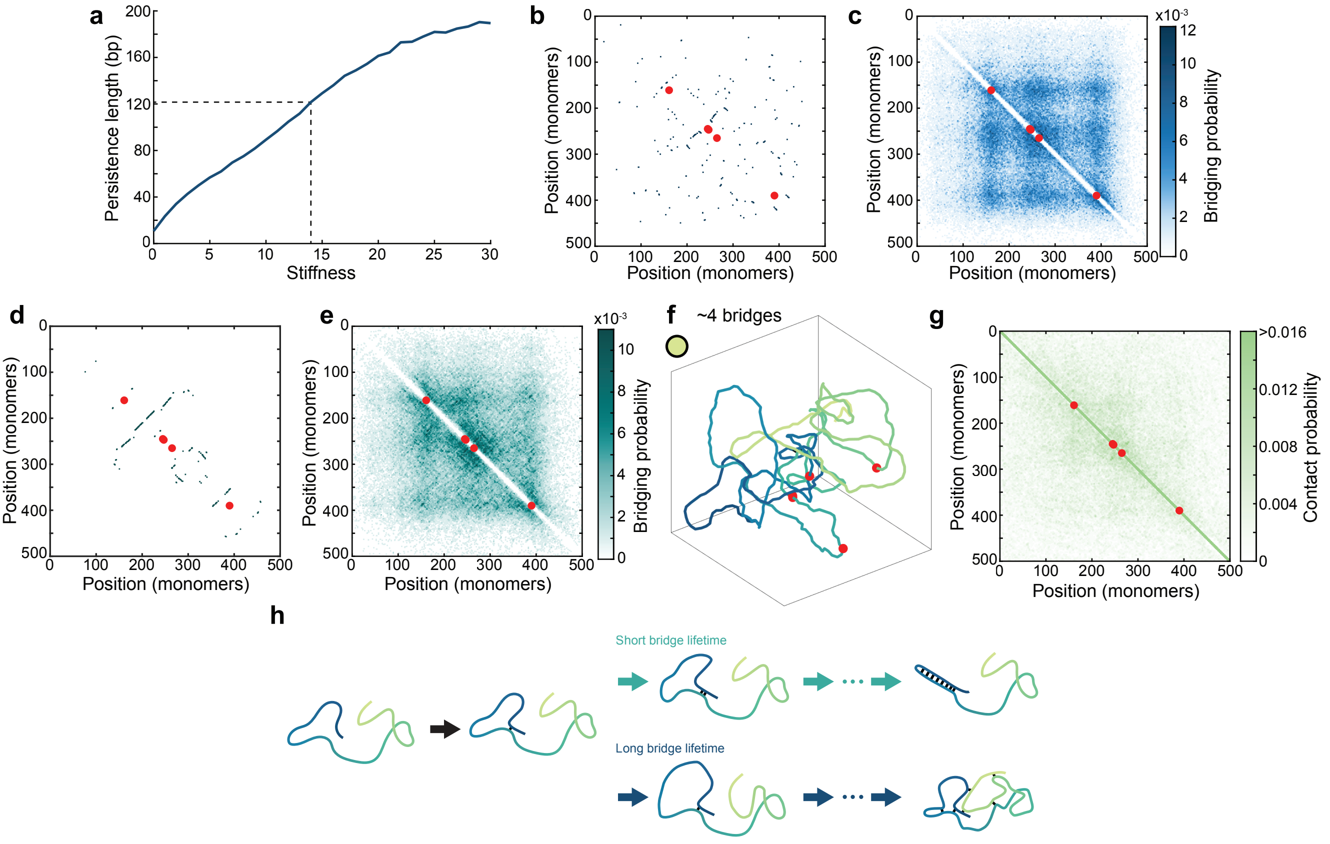

Supplementary Figure 1. ParB bridge lifetime results in distinctly different polymer conformations. a The stiffness of the polymer versus the persistence length calculated. We choose a stiffness of 14 in order to give a persistence length of ∼120 bp. **b** Individual bridge map for the globular polymer conformation shown in Figure 1f. **c** Average bridge map for the polymer in the globular regime. **d** Individual bridge map for the structured polymer conformation shown in Figure 1h. **e** Average bridge map for the polymer in the structured regime. **f** An example conformation of the polymer in the free coil-like state. The location on the phase diagram in Figure 1d is marked with a light green dot. **g** Average contact map at the same location. Based on 1000 conformations. **h** Cartoon illustrating bridging at both short and long ParB bridge lifetimes. At short bridge lifetimes the polymer conformation remains mostly unchanged and therefore bridges form close to each other. This reduces the entropic cost from the distortions caused by bridges. At long bridge lifetimes the polymer conformation can change significantly between bridging events, other genomically distant regions of the polymer may come into close contact and bridges can form between these regions. Here long and short bridge lifetimes refers to the move rate of the polymer. Source data are provided as a Source Data file.

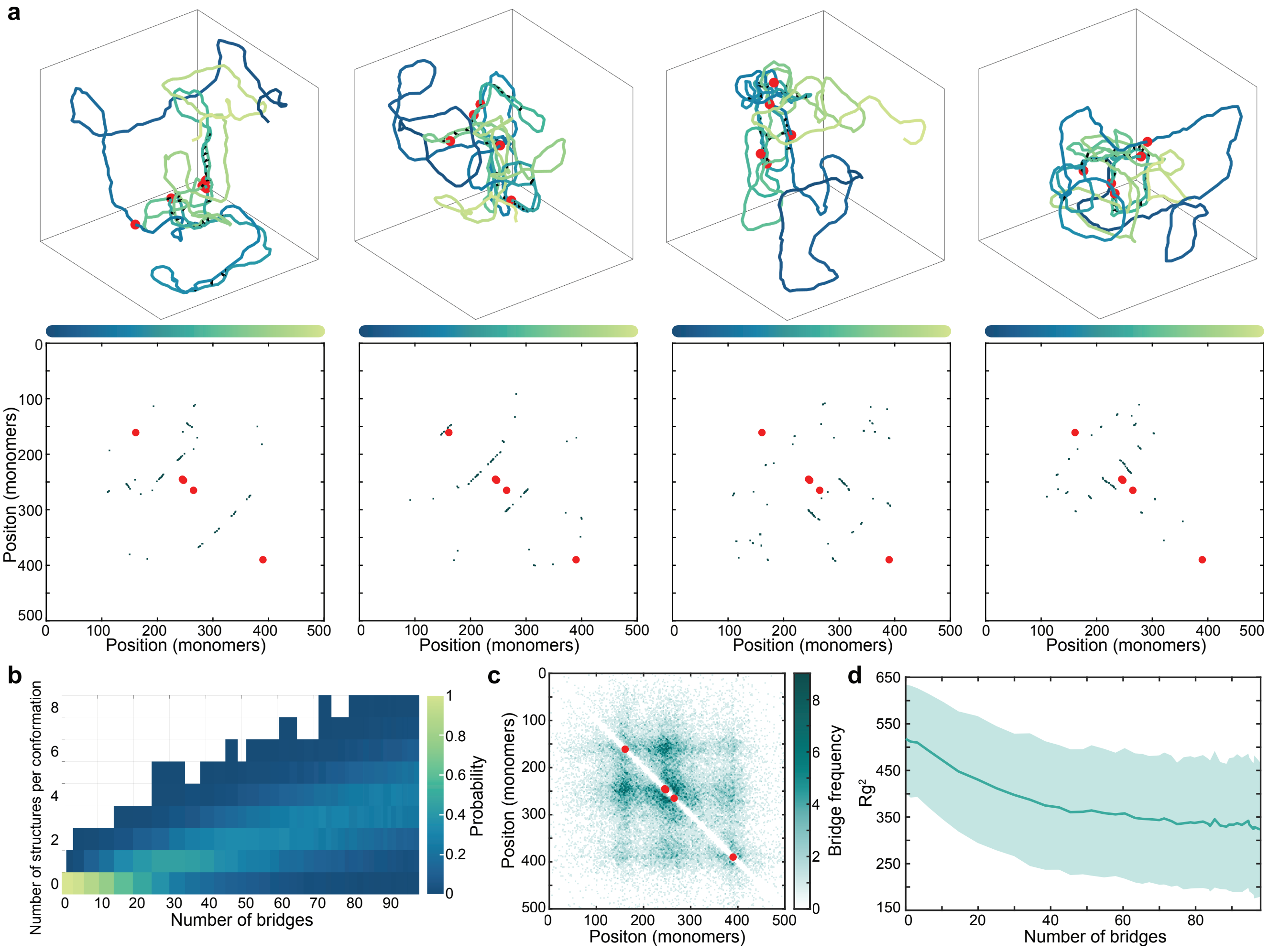

Supplementary Figure 2. Short-lived ParB bridges result in the formation of hairpins and helices. **a** Complete polymer conformations and bridge maps for the structures shown in Figure 2a. **b** Histogram showing the probability of a certain number of structures being present in any given conformation. After ~ 30 bridges it becomes most likely that a given conformation will have at least one structure. **c** Bridge map for the polymer at a population level with an average of 30 short-lived bridges. **d** Mean squared radius of gyration for the polymer as the number of bridges increases. Shading represents the SD. Source data are provided as a Source Data file.

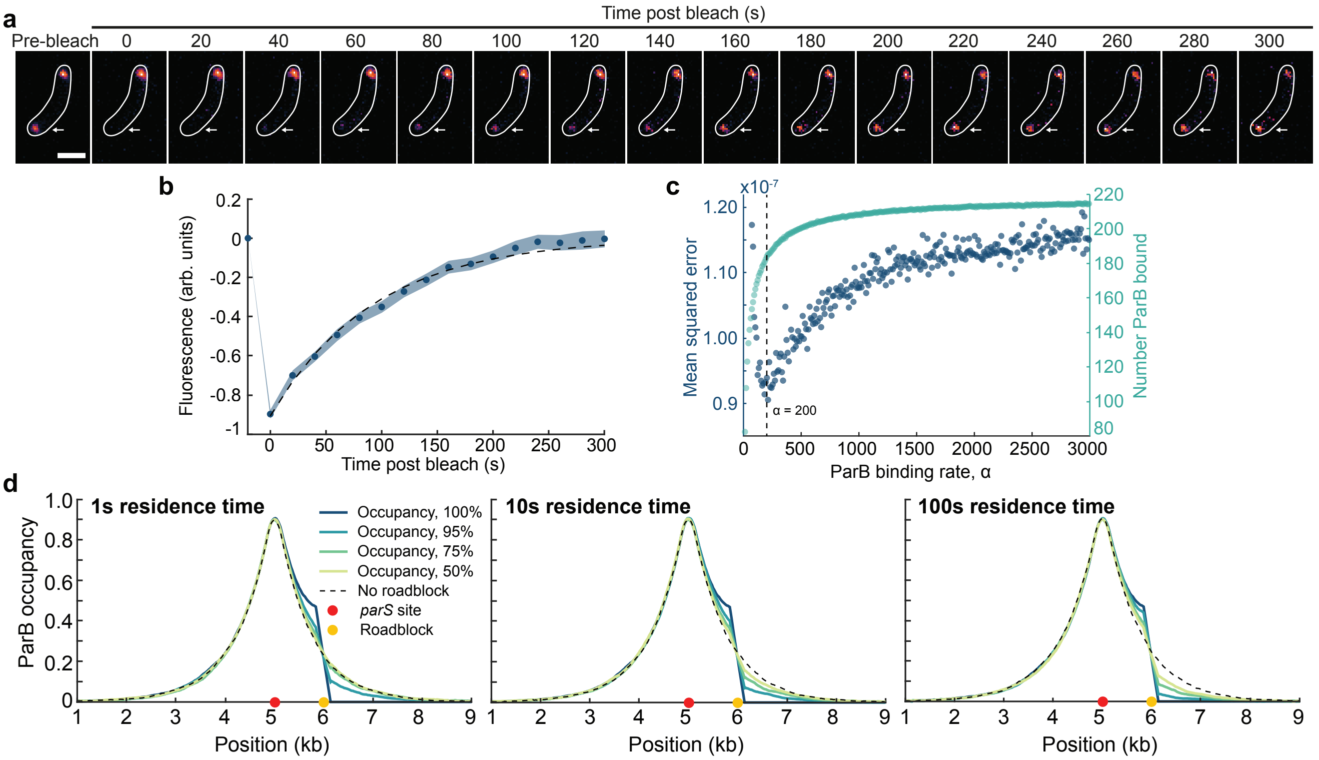

Supplementary Figure 3. ParB sliding can reproduce the multi-peaked *C. crescentus* profile. **a** Representative images of a fluorescence recovery after photobleaching experiment. A single eGFP-ParB partition complex (arrow) was photobleached in a cell containing two condensates. Scale bar is 1 µm. **b** Difference curve $B_{-}(t) = B_1(t) - B_2(t)$ (blue) as described in FRAP analysis methods with corresponding fitted curve (grey) finding a half life of 64 s for ParB dimers. Shading represents the SEM. Source data are provided as a Source Data file. **c** Mean squared error for the result of simulations compared to ChIP-seq data and the number of ParB bound as the ParB binding rate is varied. Grey line marks the selected ParB binding rate, $k_{\mathrm{on}} = 200\,\mathrm{s}^{-1}$. **d** Sliding profile from simulations for ParB sliding from a single *parS* site (red dot) with a roadblock located at the yellow dot where the percentage occupancy of the roadblock is varied. The residence time is varied as 1s, 10s, and 100s and displayed in each case. Source data are provided as a Source Data file.

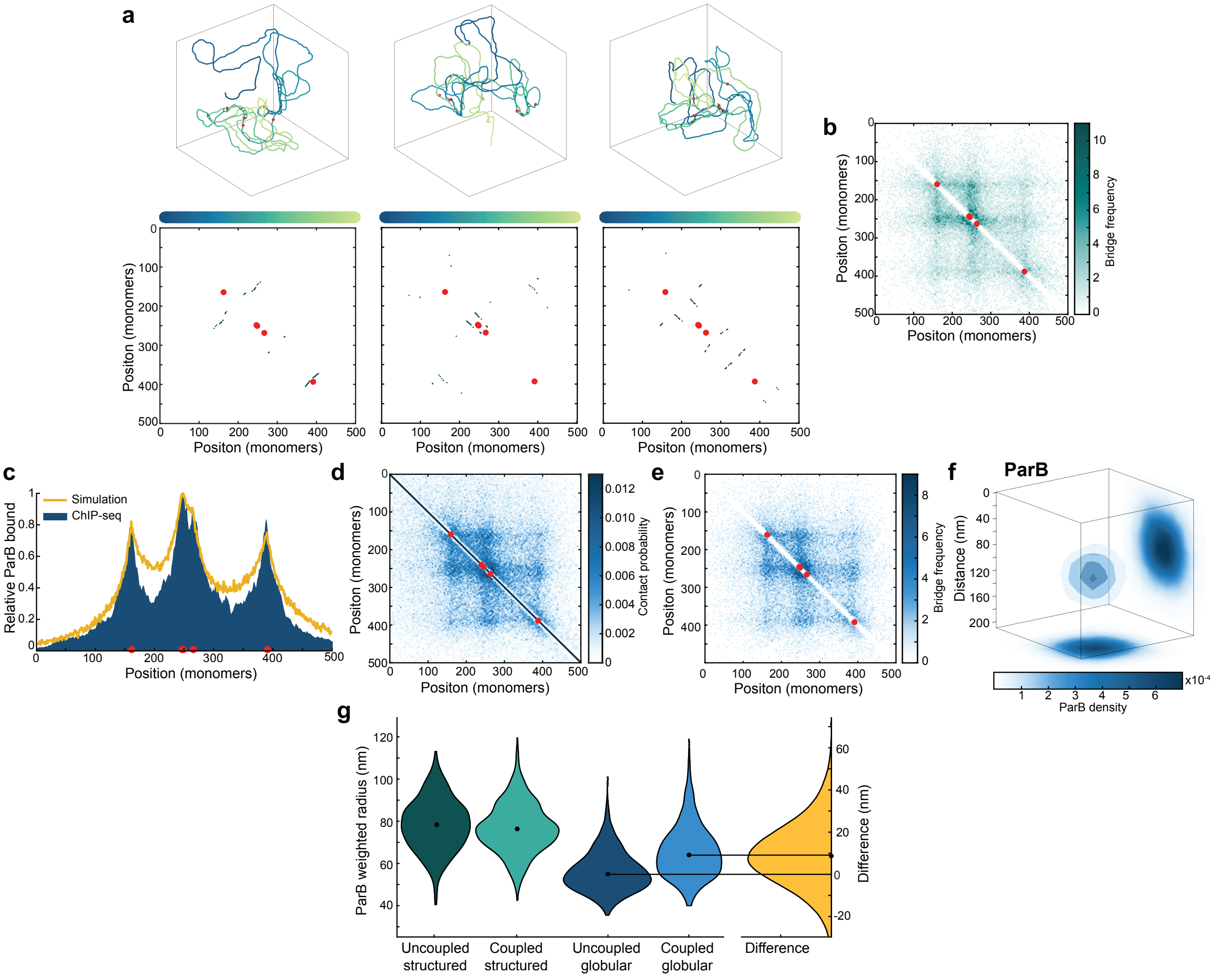

Supplementary Figure 4. ParB sliding is not inhibited by short-lived ParB bridges. a Complete conformations and individual bridge maps for hairpin and helical structures found in coupled simulations in the structured regime with an average of 25 bridges. **b** Average bridge map from coupled simulations in the structured regime with an average of 25 bridges. **c** Profile of ParB as generated from the sliding and bridging simulations in the globular regime compared to the ChIP-seq data from a previous study [1]. **d** Average contact map for coupled simulations in the globular regime with an average of 28 bridges. **e** Average bridge map from coupled simulations in the globular regime with an average of 28 bridges. **f** Three-dimensional average of the ParB partition complex in the globular state. **g** Violin difference plots for the ParB weighted radius in uncoupled and coupled (with explicit ParB dimers) simulations where there is an average of 28 bridges for both the globular and structured regimes. Source data are provided as a Source Data file.

References

[1] Hoong Chuin Lim, Ivan Vladimirovich Surovtsev, Bruno Gabriel Beltran, Fang Huang, Jörg Bewersdorf and Christine Jacobs-Wagner. Evidence for a DNA-relay mechanism in ParABS-mediated chromosome segregation. *Elife* 3, e02758 (2014).
